# Production of neutrons in laminated barriers of radiotherapy rooms: comparison between the analytical methodology and Monte Carlo simulations

**DOI:** 10.1120/jacmp.v15i6.5035

**Published:** 2014-11-08

**Authors:** Gabriel Fonseca da Silva Rezende, Luiz Antonio Ribeiro da Rosa, Alessandro Facure

**Affiliations:** ^1^ Instituto de Radioproteção e Dosimetria (IRD/CNEN) Rio de Janeiro RJ Brazil

**Keywords:** Monte Carlo simulation, laminated barriers, shielding calculation

## Abstract

The necessity to build or adapt radiotherapy rooms in reduced areas leads to the search for unconventional solutions for shielding projects. In most cases, adding metals to the primary barriers is the best alternative to shield rooms properly. However, when photons with energies equal or higher than 10 MV interact with high atomic number nuclei, neutrons are ejected and may result in a radioprotection problem for both outside and inside the room. Currently, the most widely used mathematical model to estimate the neutron dose equivalents, beyond the barriers composed by concrete and metal, is applicable only in very specific conditions. Moreover, a validation work of this model had not yet been performed. In this work, the Monte Carlo code MCNPX was used to check the validity of the aforementioned mathematical model for cases of primary barriers containing steel or lead sheets, considering the existence of linear accelerators of 15 or 18 MV. The results of the study showed that over 80% of the values obtained by computational simulations revealed deviations above a factor of 2, when compared to the analytical formula. This led to the conclusion that the McGinley method cannot be considered an adequate mathematical model to describe the mentioned physical phenomenon.

PACS numbers: 87.56.bd, 02.70.Uu.

## I. INTRODUCTION

With the high demand for linear accelerators able to operate with high‐energy photon beams, the necessity of a careful review of the shielding projects for radiotherapy rooms that will house such equipments arises. A key point of the project is the study of what materials will be used in the construction of the rooms, so that they become viable both from an economic perspective and from the point of view of available space for the building. A widely used material for shielding of radiotherapy rooms is ordinary concrete. The preference for this material is due to its physical characteristics and relatively low cost. However, the exclusive use of ordinary concrete to shield rooms that will house high‐energy accelerators can produce barriers thick enough to preclude adaptations of pre‐existing rooms or even the construction of new rooms in reduced spaces. The reason for that is the large variation between tenth‐value layers (TVL) of concrete for low and high‐energy photons.^(1)^

The use of other materials, besides the ordinary concrete, to shield radiotherapy bunkers may be desirable when low‐energy devices (e.g., equipments that use Co‐60 sources or 4 MV linear accelerators) are replaced by machines that can generate photons spectra between 6 and 18 MV. The interest in the use of other materials for the construction or upgrade of primary barriers lies in the necessity to reduce its total thickness. Thus, if space is a limiting parameter, the use of high‐density materials as shielding may be necessary, reducing the thickness of the barrier and increasing the available space for the equipment and any other building element. In this case, the shielding composed by concrete and metal sheets (laminated barrier) is a good solution for confined spaces. In the construction of laminated barriers, both steel and lead are commonly used. The former offers the advantage of being inexpensive and provides good structural support. In addition, their TVL corresponds to 11.0 cm, whereas the second material is easy to handle and offers a TVL of 5.7 cm,^(2)^ ideal for areas with severe space constraints.

The drawback in the use of metallic materials for shielding is that neutrons may be ejected when these materials are subjected to irradiation with X‐rays above a certain energy threshold, which is 6.74 MV for lead and 11.20 MV for steel, as presented in Table 1.^(3)^ Therefore, when treatment rooms are designed or adapted with laminated barriers, especially with lead bricks, neutrons can be a radiological protection problem that should be properly assessed, including when linear accelerators are operating at photon energies of 10 MV.^(4)^

**Table 1:** Threshold energy for photoneutron emission in metals which may usually be found in a radiotherapy room.

*Element*	*Atomic Weight*	*Abundance (%)*	*Threshold Energy (MeV)*
	206	25.1	8.08
Pb	207	21.7	6.74
	208	52.3	7.37
Fe	54	5.8	13.40
	56	91.7	11.20
	182	26.4	8.05
	183	14.4	6.19
W	184	30.6	7.41
	186	28.4	5.75

**Figure 1:**
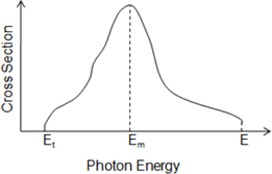
Illustration of a cross‐sectional curve for photoneutron emission.

The typical shape of the cross‐sectional curve for photoneutron emission in medium and high atomic number materials can be seen in Fig. 1. It is possible to notice that there is a rapid growth of the cross section up to a maximum value ($E_{m}$) and a gradual decrease at higher energies. As previously mentioned, there is a threshold energy ($E_{t}$) for the reaction (γ, n) to occur. For medium and heavy (A>40) nuclei, the cross‐sectional peak usually appears between 13 and 18 MV.^(5)^

Currently, the calculation of neutron doses outside the treatment rooms that contains laminated barriers is generally undertaken by the McGinley method.^6^, ^7^ According to NCRP 151,^(2)^ this method is very straightforward and covers most situations. However, it is noteworthy that a validation work of the McGinley method has not yet been extensively performed.

In this sense, the simulation codes that use the Monte Carlo method are versatile tools to study the suitability of this method, since they allow the study of several barriers configurations, using different materials. Therefore, the objective of this work is to verify the validity of the McGinley method by comparing computer simulations, using the Monte Carlo code MCNPX, and the results obtained by the analytical formula.

## II. MATERIALS AND METHODS

### A. Comparison between McGinley Method and MC simulations

In order to perform comparisons between the results from the simulations using the MCNPX code and the results obtained by McGinley's empirical formula, radiotherapy rooms with real dimensions, where the photon beam produced by the accelerator impinges on different configurations of laminated primary barriers, were simulated.

To start the model to be simulated, it was necessary to establish a procedure to calculate the thicknesses of different configurations of primary barriers. With this purpose, Eqs. (1), (2), (3), from NCRP 151, were used.^(2)^ The first equation gives the radiation transmission ($B_{\text{Trans}}$) required by the barrier, so that the dose rate outside the treatment room does not exceed the safety limits allowed:

(1) $$B_{\text{Trans}}=\frac{H_{\text{tr}}{\left(d_{\text{prim}}\right)}^{2}}{\text{WTU}}$$

where $H_{\text{tr}}$ is the dose rate limit (Sv/wk); $d_{\text{prim}}$ is the distance from X‐ray target to the point to be protected, typically between 4 and 6 m; *W* is the workload, given at the isocenter (cGy/wk); *T* is the occupancy factor, which takes into account the occupation of the area to be protected; and *U* is the use factor, the fraction of the beam‐on time that the beam is directed to each of the primary barriers.

The thicknesses of the barriers are then calculated by applying the values of tenth‐value layers, based on the photon energy and the type of the barrier material. In this case the number of TVL is given by:

(2) $$n=-\text{log}(B_{\text{Trans}})$$

For typical values of $d_{\text{prim}}$ distances, *n* ranges between 4 and 6, and the thickness L of the barrier is given by:

(3) $$L=\text{TV}L_{1}+(n-1)\text{TV}L_{e}$$

where ${\text{TVL}}_{1}$ is the first tenth‐value layer, and the equilibrium tenth‐value layer (${\text{TVL}}_{e}$) takes into account changes in the radiation spectrum due to interactions of the incident beam within the barrier.

In the case of laminated barriers, the total transmission of the photon beam produced by a linear accelerator is the product of the transmission of each individual material in the barrier — for example, for concrete, steel, and lead.^(2)^ However, this approach does not consider that the interaction process of photons with the barrier will cause photoneutron emission.

Currently, the most widely used equation in the literature for calculating the neutron dose beyond the laminated barriers is given by McGinley:^6^, ^7^

(4) $$H_{n}=\left(\frac{D_{0}R\;F_{\text{max}}}{\frac{t_{m}}{2}+t_{2}+0.3}\right)\left[10^{-\left(\frac{t_{1}}{\text{TV}L_{X}}\right)}\right]\left[10^{-\left(\frac{t_{2}}{\text{TV}L_{n}}\right)}\right]$$

where $H_{n}$ is the neutron ambient dose equivalent per week $(\mu \text{Sv}.{\text{week}}^{-1}),D_{0}$ is the X‐ray– absorbed dose per week at the isocenter $(\text{cGy}.{\text{week}}^{-1}),R$ is the neutron production coefficient $(\mu \text{Sv}.{\text{cGy}}^{-1}.m^{-2}),F_{\text{max}}$ is the maximum area of the field size at the isocenter $(m^{2}),t_{m}$ is the thickness of the metal sheet (m), $t_{1}$ is the thickness of the first concrete layer (m), $t_{2}$ is the thickness of the second concrete layer (m), *0.3* is the distance in meters from the external surface of the barrier to the point of occupation, ${\text{TVL}}_{x}$ is the concrete tenth‐value layer (m) to the primary X‐ray beam (44 cm and 45 cm, for 15 MV and 18 MV, respectively), and ${\text{TVL}}_{n}$ is the concrete tenth‐value layer to the spectrum of neutrons ejected from the metal sheet (25 cm, for both beam energies).

The coefficient values for R available in the literature were measured by McGinley^(2)^ for steel and lead, considering 15 and 18 MV photon spectra. The values obtained from measurements in 18 MV accelerators were 19 and 1.7 $\mu \text{Sv}.{\text{cGy}}^{-1}.m^{-2}$ for lead and steel, respectively, whereas the value of R decreases to $3.5\;\mu \text{Sv}.{\text{cGy}}^{-1}.m^{-2}$ for lead, considering 15 MV accelerators.

In this work, the MCNPX code (version 2.7.0) was used in order to simulate the physical processes involved in the different settings of the treatment rooms, which are provided in the modeling that will be addressed below. The MCNP (Monte Carlo N Particle) is an internationally recognized code that performs single or coupled transport of photons, electrons and neutrons. Such code uses a Cartesian coordinate system in three dimensions (x, y, z) that allows the user to adjust the source characteristics and specify the materials involved in the model, as well as select the physical quantity to be estimated.^(8)^

For the calculations presented in this work, a divergent radiotherapy beam, with the collimator set to its maximum aperture, producing a $40\times 40\;{\text{cm}}^{2}$ field (F) at the isocenter, was considered. The distance from the isocenter to the inner face of the primary barrier was set to be 4 m. Two detectors were positioned, one at the isocenter to assess the X‐rays dose $\left(D_{x}\right)$, and another at 30 cm from the outer face of the primary barrier (in the same direction of the photon beam) to estimate the neutron dose equivalent ($H_{n}$). The photon spectra were chosen as 15 MV and 18 MV, according to the description provided by Sheikh‐Baghert and Rogers.^(9)^ The composition of the primary barrier was considered as a combination of ordinary concrete and metal sheets (steel and lead).

For calculation purposes, the metal sheets were considered positioned in three different locations: on the inner surface, in the middle, and on the outer surface of the concrete barrier. The total thickness of the barrier was set to be 4, 5, or 6 TVLs, assuming typical values for a room that houses a linear accelerator whose photons can reach 15 or 18 MV.

A description of the arrangements that were the basis for the calculations and simulations will be presented in three steps. Such arrangements are hypothetical, but can be found in some radiotherapy rooms. The fixed parameters mentioned before should be understood as a part of the all described arrangements.

**Figure 2:**
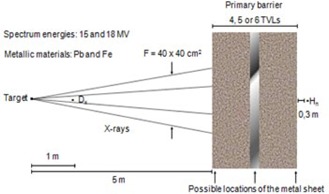
Illustration of the geometry problem (seen in profile), as well as the identification of the relevant modeling parameters.

The first stage was characterized by the combination between an 18 MV photon spectrum and sheets of lead — the metallic material composing the barrier. The thicknesses of the metallic sheets in this step were set to 5.7 cm and 11.4 cm, corresponding to one and two TVLs, respectively. In the second step, the metal element used was the same, but the 15 MV photon spectrum was taken in account. In this step, the selected thickness for the lead sheet was 11.4 cm. Finally, the third step was set to an 18 MV photon spectrum and a laminated barrier presenting steel as metal material. In this step, the steel thickness was set to 22 cm, corresponding to two TVLs. In total, the three steps produced 36 settings, respecting the positioning of the metal sheets as well as the total thickness of the primary barrier, as illustrated in Fig. 2. It can be noticed that in steps 2 and 3, only the thicknesses corresponding to two TVLs were considered. The reason for that is the fact that thicker metal sheets compensate the lower cross sections, allowing a greater fluence of photoneutrons and hence smaller uncertainties at the detector. Altogether, 36 simulation results were compared with an equal number of results obtained with the analytical formula.

During the MCNPX simulations, the F5 tally was applied in order to provide the neutron fluences at the point detectors in X, Y, Z coordinates. Other types of tally cards (DF and DE) have been used to convert the neutron fluences (neutrons/cm^2^) to ambient dose equivalents (pSv), with the flux to dose conversion factors (pSv.cm^2^) obtained from ICRP Publication 74.^(10)^ The DE and DF cards allow modeling an energy‐dependent dose function that is a continuous function of energy, from a table whose data points need to coincide with the tally energy bin structure.

Seeking an acceptable level of uncertainties, all simulations were performed with a stopping criterion of 48 hrs. This resulted in an average number of histories in the order of $4\times 10^{7}$. Moreover, the variance reduction technique using geometry splitting with Russian roulette was implemented, in order to reduce the final uncertainty. All neutron ambient dose equivalents generated as a result of MCNPX simulations were normalized per Gray of X‐ray absorbed dose at the isocenter. Hence, a multiplying factor of $1.13\times 10^{14}$ was used to produce an absorbed dose that corresponds to 1 Gy, due to the therapeutic beam, at the point detector simulated at the isocenter (since MCNP results are normalized by the number of particles emitted from the source).

Ordinary concrete with density of $2.34\;g.{\text{cm}}^{-3}$ was used to perform the simulations. Regarding the composition of the concrete, this was based on Jaeger et al.^(11)^ (Table 2). The validation of the MCNPX simulations was already performed in another work, as presented in Facure et al.^(4)^ In this article, the authors generated a set of inputs that were executed in order to reproduce Cardman's experimental results^(12)^ for the spectrum from the decay of neutrons observed for Lead‐208 after excitation by 13.27 MeV gamma rays.

**Table 2:** Chemical composition of the ordinary concrete used in this work.

*Element*	*Density* $\left(g/{\text{cm}}^{3}\right)$	*Percentage (%)*
H	0.013	0.55
O	1.165	49.81
Na	0.040	1.71
Mg	0.006	0.26
Al	0.107	4.58
Si	0.737	31.50
S	0.003	0.13
K	0.045	1.92
Ca	0.194	8.30
Ni	0.029	1.24
Total	2.339	100

## III. RESULTS & DISCUSSION

### A. Validation of photoneutron emission with MCNPX code

**Figure 3:**
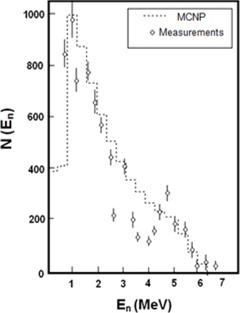
Comparison between the experimental and simulated values for the neutron emission spectrum generated by the incidence of 13.27 MeV gamma rays on sheets of Lead‐208.

From the observation of data obtained by Facure et al.^(4)^ it can be concluded that there is a good agreement between the values obtained using the MCNP code and those obtained by measurements. From Fig. 3, a superposition of both curves is observed, corresponding to a common area of 80%. The uncertainties of the simulations were below 1%.

### B. Neutron ambient dose equivalent rates beyond laminated barriers

The ambient dose equivalent rate (μSv/wk) for neutrons obtained by the McGinley method and by the MCNPX code can be found in Tables 3, 4, 5.

**Table 3:** Neutron ambient dose equivalent rates produced by a photon spectrum of 18 MV for different configurations of thickness and positioning of the lead in the primary barrier.

	*Barrier Configuration*	*McGinley Equation* (μSv/wk)	*MCNPX Results* (μSv/wk)	*Ratio (MCNPX/ McGinley)*
	1 TVL of Pb inside the room	1.30	7.69	5.9
	2 TVL of Pb inside the room	90.69	355.00	3.9
4 TVL	1 TVL of Pb middle of the barrier	25.97	134.00	5.2
	2 TVL of Pb middle of the barrier	697.65	2360.00	3.4
	1 TVL of Pb outside the room	1135.60	2480.00	2.2
	2 TVL of Pb outside the room	9433.00	22200.00	2.4
	1 TVL of Pb inside the room	a	a	a
	2 TVL of Pb inside the room	1.28	7.18	5.6
5 TVL	1 TVL of Pb middle of the barrier	a	a	a
	2 TVL of Pb middle of the barrier	25.24	78.60	3.1
	1 TVL of Pb outside the room	125.80	325.00	2.6
	2 TVL of Pb outside the room	1044.95	2270.00	2.2
	1 TVL of Pb inside the room	a	a	a
	2 TVL of Pb inside the room	a	a	a
6 TVL	1 TVL of Pb middle of the barrier	a	a	a
	2 TVL of Pb middle of the barrier	0.96	1.82	1.9
	1 TVL of Pb outside the room	13.94	16.87	1.2
	2 TVL of Pb outside the room	115.75	212.60	1.8

a Values not considered because the uncertainties in MCNPX were above 20%.

**Table 4:** Neutron ambient dose equivalent rates produced by a photon spectrum of 15 MV for different configurations of thickness and positioning of the lead in the primary barrier.

	*Barrier Configuration*	*McGinley Equation* (μSv/wk)	*MCNPX Results* (μSv/wk)	*Ratio (MCNPX/McGinley)*
	2 TVL of Pb inside the room	24.97	237.00	9.5
4 TVL	2 TVL of Pb middle of the barrier	162.38	1180.00	7.3
	2 TVL of Pb outside the room	2025.96	11000.00	5.4
	2 TVL of Pb inside the room	0.42	6.23	14.8
5 TVL	2 TVL of Pb middle of the barrier	6.82	58.30	8.5
	2 TVL of Pb outside the room	248.61	1090.00	4.4
	2 TVL of Pb inside the room	*	*	*
6 TVL	2 TVL of Pb middle of the barrier	*	*	*
	2 TVL of Pb outside the room	30.51	137.00	4.5

**Table 5:** Neutron ambient dose equivalent rates produced by a photon spectrum of 18 MV for different configurations of thickness and positioning of the steel in the primary barrier.

	*Barrier Configuration*	*McGinley Equation* (μSv/wk)	*MCNPX Results* (μSv/wk)	*Ratio (MCNPX/ McGinley)*
	2 TVL of Fe inside the room	7.78	23.90	3.1
4 TVL	2 TVL of Fe middle of the barrier	58.53	124.00	2.1
	2 TVL of Fe outside the room	734.90	3260.00	4.4
	2 TVL of Fe inside the room	*	*	*
5 TVL	2 TVL of Fe middle of the barrier	2.15	3.81	1.8
	2 TVL of Fe outside the room	81.41	359.00	4.4
	2 TVL of Fe inside the room	*	*	*
6 TVL	2 TVL of Fe middle of the barrier	*	*	*
	2 TVL of Fe outside the room	9.02	27.30	3.0

Therefore, in order to obtain the ambient dose equivalent rate in μSv/wk, it was necessary to multiply each result from the MCNPX by the accelerator weekly workload, which was typically considered as 10^5^ cGy/wk.

According to the results shown in Tables 3, 4, 5, it can be seen that the comparison between MCNPX simulations and the analytical formula reveal discrepancies that range from a factor of 1.2 to 14.8. Analyzing the differences between the values resulting from the aforementioned equation and MCNPX, it was found that over 80% of the values obtained by computational simulations showed deviations above a factor of 2, when compared to the analytical formula.

A direct observation, from the data presented in Tables 3, 4, 5, is that the results obtained from the simulations are always higher than those obtained by the analytical equation. This fact discourages the use of this equation, due to the aspects of radioprotection. Furthermore, a fixed distance of 4 m between the isocenter and the inner surface of the primary barrier was considered, representing a value that is commonly found in radiotherapy rooms.

It was noticed that McGinley's equation is very sensitive to the ${\text{TVL}}_{n}$ parameter, which can be one of the major causes of discrepancy. Therefore, ${\text{TVL}}_{n}$ values were obtained, taking in account each particular barrier configuration. It was found that, for the 18 MV spectrum, this parameter ranges from 29.0 to 35.8 cm, depending on the barrier configuration. However, to be conservative and stay on the safe side, the utilization of a ${\text{TVL}}_{n}$ value of 36 cm in McGinley's equation is recommended. For the cases where the barriers configuration are similar to those presented in this article, the ratios presented in Tables 3, 4, 5 can be directly applied to provide a correction of McGinley's equation.

The MCNPX results that were considered valid for the comparison with the results of the studied equation are those whose uncertainties were below 20% — what is considered acceptable, for example, in environmental monitoring.

## IV. CONCLUSIONS

This study aimed to verify the validity of the McGinley method by simulations using the Monte Carlo code MCNPX. This study was motivated by the absence of a extensively validation work for the method mentioned above, as well as by the lack of methods to describe the physical problem presented in the body of this paper.

The simulations obtained in this work showed that the neutron ambient dose equivalents rate differs from a factor of 1.2 to 14.8 in comparison with the results calculated using the analytical methodology. Also, it was found that all the simulated values are higher than the values calculated by McGinley's equation, which is undesirable from the point of view of radiation protection.

The suggestion of using 36 cm as the ${\text{TVL}}_{n}$ parameter in McGinley's equation produces more conservative results accordingly to aspects of radiation protection. Moreover, the ratios presented in Tables 3, 4, 5 can be directly applied as correction factors for the analytical methodology, when the studied barrier configuration is similar to the ones studied in this article. For different and more complex cases, the use of other calculation methods is strongly recommended, as personalized Monte Carlo studies.
